# Late onset neonatal sepsis: Can plasma gelsolin be a promising diagnostic marker?

**DOI:** 10.1097/MD.0000000000037356

**Published:** 2024-03-08

**Authors:** Wesam A. Mokhtar, Laila M. Sherief, Naglaa M. Kamal, Azza O. ElSheikh, Farida H. Omran, Ahmed Abdulsaboor, Maha M.H. Sakr, Shreif El Gebally, Mohamed M. M. Shehab, Jaber Alfaifi, Reem Turkistani, Futun Aljuaid, Mohammed A.M. Oshi, Fouad B.A. Elbekoushi, Ghada A. Mokhtar

**Affiliations:** a Pediatric Department, Faculty of Medicine, Zagazig University, Zagazig, Egypt; b Pediatric Department, Faculty of Medicine, Cairo University, Giza, Egypt; c Medical Microbiology and Immunology Department, Faculty of Medicine, Zagazig University, Zagazig, Egypt; d Clinical Pathology Department, Faculty of Medicine, Zagazig University, Zagazig, Egypt; e Department of Child Health, Faculty of Medicine, University of Bisha, Bisha, Kingdom of Saudi Arabia; f Pediatric Department, Alhada Armed Forces Hospital, Taif, Kingdom of Saudi Arabia; g Pediatric Department, Taif Children Hospital, Taif, Kingdom of Saudi Arabia; h Neurology Division, Pediatric Department, Gaafar Ibnauf Children’s Emergency Hospital, Khartoum, Sudan; i Pediatric Department, Zagazig University, Zagazig, Egypt.

**Keywords:** gelsolin, late-onset sepsis, marker, neonate

## Abstract

Plasma gelsolin (pGSN) correlates with clinical improvement in septic patients. We aimed to investigate pGSN levels as a diagnostic and prognostic marker of neonatal late-onset-sepsis (LOS). A case-control study was done on 184 neonates (92 with LOS and 92 controls). All participants were subjected to detailed history taking, full clinical evaluation, sepsis workup, and pGSN enzyme-linked immunosorbent-assay measurement. We detected significantly lower pGSN level among cases compared to controls (90.63 ± 20.64 vs 451.83 ± 209.59). It was significantly related to the severity of sepsis and mortality, with significantly lower values among cases with septic shock and multiorgan failure and non-survivors. Follow-up pGSN significantly increased after sepsis improvement in survivors compared to admission values. pGSN might be a reliable diagnostic and prognostic marker for LOS.

## 1. Introduction

Neonatal sepsis is a clinical syndrome of bacteremia with systemic signs and symptoms of infection in the first 4 weeks of life usually, without localization (septicemia) and sometimes with localization to the lungs or the meninges.^[1]^

The hemodynamic instability and/or respiratory failure are usually late signs in neonates. Neonatal sepsis is either early or late onset. The early-onset sepsis is that within the 1st 3 to 7 days of life while the late-onset sepsis (LOS) occurs beyond that.^[2]^

Sepsis is the most important cause of death in critically ill patients. Delays in its diagnosis and of antibiotics initiation have been shown to increase mortality. However, differentiating sepsis from noninfectious triggers of the systemic inflammatory response syndrome is difficult.^[3]^

Gelsolin is a calcium-dependent actin-binding protein. It has a protective role against tissue injury. Actin has a major role in the settlement of dynamics, motion of cells, process of phagocytosis, regulation of apoptosis, modulation of thrombocyte, as well as anti-inflammatory function.^[4]^

Gelsolin is available both intracellularly (cytoplasmic) as well as secreted extracellularly (plasma). Plasma gelsolin (pGSN) is secreted in high amounts in skeletal muscles and is present in blood at high levels. Furthermore, it is found to be lower by 20% to 50% in several clinical conditions.^[5]^

Studies of sepsis have shown that decreased pGSN correlates with elevated circulating levels of actin secreted by the cells during inflammation and infection. Moreover, pGSN changes correlate with clinical improvement in septic patients.^[6]^

we aim to investigate the role of pGSN levels as a diagnostic and prognostic marker of late onset neonatal sepsis.

## 2. Patients and methods

A case-control prospective study was carried over a period of 12 months in the neonatal intensive care unit (NICU) of Zagazig University Children Hospital, Zagazig University, Egypt. Ninety-two full-term neonates fulfilling the study inclusion criteria were enrolled in the study. Another 92, age and sex-matched neonates with no evidence of neonatal sepsis were recruited as controls.

*Inclusion criteria* included neonates diagnosed as LOS by clinical assessment and confirmed by positive blood culture, with age ≤ 28 days and gestational age: >37 to <42 weeks. No gender restrictions.

*Exclusion criteria* included those with multiple congenital anomalies, inborn errors of metabolism, postoperative patients, patients with underlying serious cardiac problems, hypoxic-ischemic encephalopathy, or intracranial hemorrhage. Patients with early-onset sepsis were excluded.

All participants were subjected to full history taking and detailed clinical assessment including maternal history (disease, infection, drug intake, etc), natal history (mode of delivery, outcome of pregnancy, needs of resuscitation), post-natal history, family history, estimation of gestational age, anthropometrics measurement, vital signs, detailed systemic examination, and head, neck, back and genitalia examination.

All participants were subjected to routine laboratory investigations according to NICU protocols including sepsis work-up (i.e., complete blood count with differential cell count and immature to total neutrophil ratio, C-reactive protein level, procalcitonin and blood culture).

Neonates with LOS were classified according to the severity of sepsis:

1. Sepsis: Those who had manifestations of SIRS and proven infection.
2. Severe sepsis: Those who had manifestations of sepsis, organ dysfunction, hypoperfusion, or hypotension.
3. Septic shock and multiorgan failure (MOF): Those who had cardiovascular dysfunction including persistent hypotension despite adequate fluid replacement, need for a vasopressor to sustain blood pressure and the existence of altered organ function in severely ill patients.^[7]^

Patients were strictly followed up during their NICU stay regarding the clinical course, recommended antibiotics, the negativity of sepsis markers, and the final outcome.

Both patients and controls were subjected to measuring pGSN levels. Another blood sample for follow up pGSN (FU-pGSN) detection was obtained after 10 days of antibiotic administration in survived cases.

## 3. Methods

### 3.1. Blood sample

Two mL of peripheral venous blood were immediately withdrawn for all participants on admission and after 10 days of antibiotic use only in survived cases. The samples were placed in a sterile EDTA-containing test tube and centrifuged at 155 × g for 10 minutes at 4°C to collect plasma. The plasma was stored at −80°C until analysis.

## 4. Measuring pGSN level by enzyme-linked immunosorbent assay

The pGSN levels were measured using Human Gelsolin enzyme-linked immunosorbent assay Kits (MyBioSource Inc., San Diego, CA, USA) according to manufacturer instructions. The results were expressed in ng/mL.

## 5. Statistical analysis

The collected data were analyzed using Statistical Package for Social Science (SPSS) version 24. Qualitative data were presented as numbers and percentages, and quantitative data were expressed as mean ± standard deviation and range. Chi-square test (χ^2^) was used to analyze qualitative variables, while the Student *t* test (*t*) and One-Way Analysis of variance (ANOVA) test were used to analyze continuous data. Pearson correlation coefficient (*r*) was used to test the correlation between continuous variables. Paired *t* test for the dependent sample was used to compare 2 variables in the same group. The results were considered statistically significant and highly statistically significant when the significant probability (*P* value) was < .05 and < .001, respectively.

## 6. Results

A total of 184 neonates were enrolled in the current study. They were divided into 2 groups; cases: which included 92 neonates with LOS and controls: which included 92 neonates without sepsis. Demographic data of both groups were equal at the time of enrollment (*P* ≥ .05) (Table 1).

**Table 1:** Demographic of the studied groups.

Parameter			Cases (n = 92)	Controls (n = 92)	Test	*P* value
Gestational age (wk)	Mean ± SD		38.02 ± 1.62	37.89 ± 1.61	0.387*	.700
	Range		37–41	37–41		
Age on admission (d)	Mean ± SD		15.63 ± 4.75	15.91 ± 4.85	−0.282*	.779
	Range		7–25	7–28		
Weight (kg)	Mean ± SD		3.54 ± 0.33	3.55 ± 0.35	0.150*	.881
	Range		2.9–4.3	2.8–4.5		
Sex	Female	N	40	48	0.697†	.404
		%	43.5%	52.2%		
	Male	N	52	44		
		%	56.5%	47.8%		
Mode of delivery	Normal vaginal delivery	N	36	28	0.766†	.381
		%	39.1%	30.4%		
	Caesarean section	N	56	64		
		%	60.9%	69.6%		

SD = standard deviation.

* Student *t* test.

† Chi square test.

Cases were classified according to the severity of sepsis into; those with sepsis (n = 26), severe sepsis (n = 40), and septic shock with MOF (n = 26).

There was a significant statistical difference between cases and controls regarding initial laboratory investigation (total leukocytic count (*P* < .05), immature/total neutrophil ratio (*P* < .001), hemoglobin (*P* < .001), platelet (*P* < .001), C-reactive protein (*P* < .001), and procalcitonin (*P* < .001).

The most common initial clinical presentation of patients was decreased activity (84.8%) (Table 2). Positive blood cultures revealed the following organisms; *Staphylococcus haemolyticus* (39.1%), *Staphylococcus hominis* (21.7%), *Klebsiella pneumonia* (17.4%), *Pseudomonas aeruginosa* (8.7%), *Escherichia coli* (8.7%), and *Enterococcus faecalis* (4.4%).

**Table 2:** Initial clinical presentations of the studied cases.

Variables	n = 92	%
Initial clinical presentation		
Decreased activity	78	84.8
Respiratory distress	68	73.9
Apnea	18	19.6
Temperature instability	72	78.3
Neurological manifestation	6	6.5
Gastrointestinal manifestations	56	60.9
Hepatosplenomegaly	10	10.9
Pneumonia	48	52.2
Myocarditis	4	4.3
Hematological manifestation	36	39.1

The level of pGSN level was significantly lower in cases (76 μg/mL) as compared to controls (249 μg/mL) with *P* < .001 and range of 65 to 98 and 164.7 to 324.3 μg/mL respectively.

Moreover, among cases, pGSN was significantly lower (*P* < .001) in patients with septic shock and MOF [59 (56.5–70.5) μg/mL] than in those with sepsis [101 (96–104) μg/mL] and severe sepsis [75.5 (65–94) μg/mL]. pGSN level was found to be associated with patients’ outcome as it was significantly lower (*P* < .001) in nonsurvivors [65 (57–71.5) μg/mL] as compared to survivors [87 (71–101.5) μg/mL].

**Figure 1.**
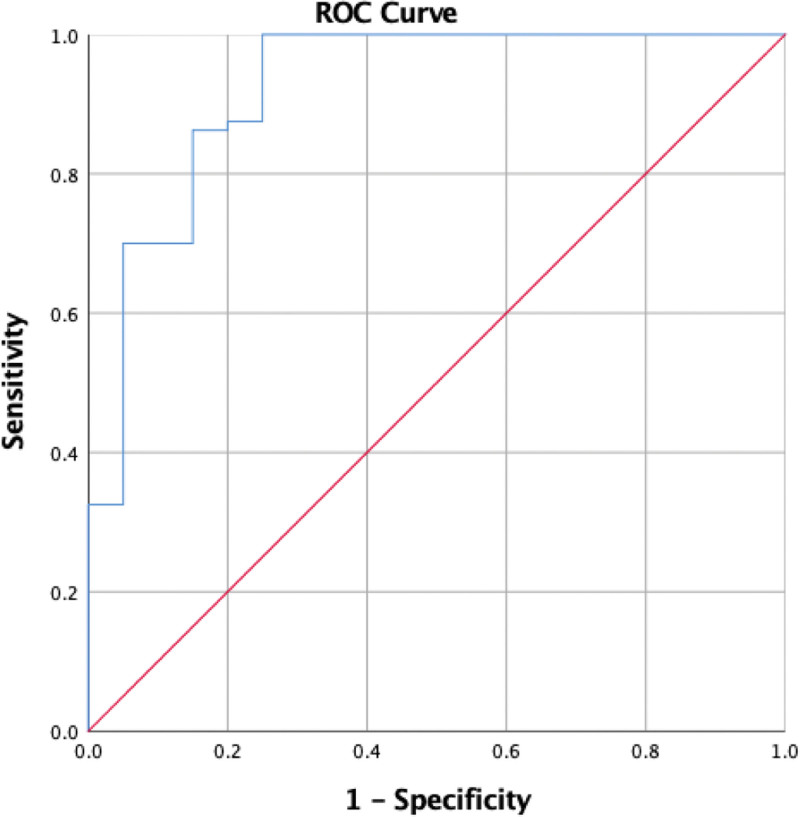
ROC curve for pGSN as a predictor for mortality. N.B: Area under the curve is 0.923. Best cut off is 57.1 with 100% sensitivity 100% and 55% specificity. pGSN = plasma gelsolin.

A cut off of ≤ 57.1 μg/mL was found to be the best predictor of mortality with 100% sensitivity and 55% specificity (Fig. 1).

When patients started to improve clinically, FU-pGSN started to increase. FU-pGSN increased significantly [205 (163–293) μg/mL] as compared to its levels at the time of enrollment [87 (71–101.5) μg/mL] (*P* < .001). Hence, pGSN level can be a good marker of improvement in survivors.

## 7. Discussion

Neonatal sepsis is considered one of the life-threatening conditions in the first 28 days of life. It is an infection involving the bloodstream of neonates and is mainly caused by bacteria.^[8]^ Due to the nonspecific clinical symptom, the diagnosis of neonatal sepsis often presents a high false negative rate and a delay in obtaining blood culture results. Thus, biomarkers for distinguishing sepsis accurately at earlier stages are urgently needed.^[9]^ The early diagnosis and timely management of sepsis are known to be crucial in the reduction of sepsis-induced mortality.^[10]^

Many studies highlighted that association between decreased pGSN levels and the severity and prognosis of different diseases, such as bronchopulmonary dysplasia, hyperoxic lung injury, sepsis, and intracranial hemorrhage.^[11]^ We herein aim to assess the role of pGSN as a diagnostic and prognostic marker of late onset neonatal sepsis in neonates.

Regarding the bacteria isolates, searching Egyptian data revealed that, Shehab El-Din et al^[12]^ conducted a study on a total of 357 neonates with suspected sepsis recruited from 3 NICUs in Mansoura city, in which, about 91 neonates showed positive blood cultures. The bacterial isolates revealed coagulase-negative staphylococci (46.15%) followed by *K pneumonia* (19.78%). Furthermore, Mohsen et al^[13]^ studied the prevalence of early and LOS in NICU at Cairo University Children Hospital. From a total of 191 LOS neonates, 117 showed positive culture results. *K pneumonia* (41.9%) was the most common bacterial isolate followed by CONS (13.7%), *P aeruginosa* (11.9%), *Acinetobacter* (8.5%), Methicillin resistance *Staphylococcus aureus* (6%), *Enterobacter* (6%), *E Coli* (3.4%), and *B streptococci* (3.4%). Moreover, some authors stated that *K pneumonia* was the most commonly encountered bacterial isolate in NICU patients.^[14,15]^ The diversity of organisms supports the fact that organisms causing sepsis vary from one region to another and change over time even in the same place.

Our study showed that there was a statistically significant decrease in pGSN levels in cases when compared to controls (median (IQR) of 76 (65–98) vs 249 (164.7–324.3) μg/mL). In accordance with our results, studies in adults with sepsis revealed that the mean value of pGSN was significantly lower in cases than in controls.^[16–18]^ Halis et al^[19]^ in their study in preterm infants with sepsis reported similar results. They found that the mean pGSN level at the time of diagnosis in the sepsis group was 33.98 ± 11.44 μg/mL, which was significantly lower than that of the control group (60.05 ± 11.3 μg/mL).

Regarding the relation between pGSN and sepsis severity, we demonstrated a significant statistical decrease in pGSN levels among neonates with septic shock and MOF (*P* < .001) as compared to other sepsis groups. Similar data were detected in adult studies.^[18]^ El-Akabawy et al^[18]^ classified critically ill septic adult patients into those with sepsis (n = 25), severe sepsis (n = 21), and septic shock (n = 34). They found a significant decrease in pGSN level with increased severity of sepsis (*P* = .009).^[18]^ Similarly, Horváth-Szalai et al^[20]^ found that pGSN levels were lower in patients with severe sepsis as compared to those with sepsis [16.74 (8.21–34.57) and 26.31 (16.89–28.25) μg/mL, respectively].

Out of the total 92 septic neonates included in our study, 33 (71.7%) survived and 13 (28.3%) died. Lower pGSN level significantly correlated with mortality due to sepsis. There was a significant statistical decrease in pGSN level among nonsurvivors [median (IQR) of 65 (57–71.5)] in relation to survivors [median (IQR) of 87 (71–101.5) μg/mL] with a *P* value of < .001. A cutoff of ≤ 57.1 μg/mL was found to be the best predictor of mortality.

In correspondence with the above results, Wang et al^[16]^ demonstrated decreased pGSN levels among the nonsurvivors septic critically ill surgical adult patients. Additionally, Lee et al^[17]^ reported significantly low pGSN levels among the nonsurvivors of septic nonsurgical adults compared to survivors (mean ± standard deviation of 89 ± 48 vs163 ± 47 μg/mL, *P* = .01). Among preterm neonates, Halis et al,^[19]^ reported that pGSN level was higher in survivors than non-survivors but was not significant statistically (*P* = .74), this difference might be due to the difference in maturity/gestational age (term neonates in our study/versus preterm neonates in their study.

Accordingly, it can be concluded that the pGSN level might be used as a predictor of sepsis severity and sepsis-associated mortality.

In the current work, we reevaluated pGSN levels among survivors after clinical improvement and completing 10 days antibiotics course, where we found a statistically significant increase in FU-pGSN levels [median (IQR) of 87 (71–101.5) vs 205 (163–293) μg/mL, *P* < .001]. Wang et al^[16]^ found similar results in their adult cohort. Similarly, Halis et al,^[19]^ demonstrated the same in their cohort of septic preterm neonates.

An ideal biomarker should be easily obtainable, has rapid detection, and reflects normal and pathogenic processes through quantitative and reproducible estimation.^[21]^ pGSN can fulfill most pGSN can fulfill most of those criteria and accordingly might be a reliable diagnostic and prognostic biomarker for neonatal sepsis.

## 8. Limitations

The present study had some limitations being a single center study with a relatively small sample size. Another limitation is the scarcity of the studies carried in neonates with LOS. Larger cohort multicenter studies are warranted.

## 9. Conclusions

Significantly lower pGSN levels were detected among septic neonates and neonates with septic shock and MOF than those with less severe sepsis. Moreover, it is found to be significantly related to sepsis, mortality, and prognosis. Thus, it might be valuable as a reliable diagnosis and prognostic marker for neonatal sepsis. Further studies on larger scales and in different geographical areas are recommended.

## Author contributions

**Conceptualization:** Wesam A. Mokhtar, Laila M. Sherief, Fouad B.A. Elbekoushi.

**Data curation:** Wesam A. Mokhtar, Laila M. Sherief, Shreif El Gebally, Fouad B.A. Elbekoushi.

**Formal analysis:** Wesam A. Mokhtar, Farida H. Omran, Azza O. Elsheikh, Fouad B.A. Elbekoushi, Ahmed Abdulsaboor, Maha M.H. Sakr.

**Investigation:** Wesam A. Mokhtar, Ahmed Abdulsaboor, Maha M.H. Sakr, Shreif El Gebally, Ghada A. Mokhtar.

**Methodology:** Wesam A. Mokhtar, Ahmed Abdulsaboor, Maha M.H. Sakr, Shreif El Gebally, Mohamed M. M. Shehab, Ghada A. Mokhtar.

**Project administration:** Wesam A. Mokhtar, Ahmed Abdulsaboor, Maha M.H. Sakr, Mohamed M. M. Shehab, Fouad B.A. Elbekoushi.

**Supervision:** Wesam A. Mokhtar.

**Validation:** Ahmed Abdulsaboor, Maha M.H. Sakr.

**Visualization:** Ahmed Abdulsaboor, Maha M.H. Sakr.

**Writing – original draft:** Wesam A. Mokhtar, Laila M. Sherief, Naglaa M. Kamal, Azza O. Elsheikh, Farida H. Omran, Shreif El Gebally, Mohamed M. M. Shehab, Jaber Alfaifi, Reem Turkistani, Futun Aljuaid, Mohammed A.M. Oshi, Ahmed Abdulsaboor, Maha M.H. Sakr, Ghada A. Mokhtar.

**Writing – review & editing:** Wesam A. Mokhtar, Laila M. Sherief, Naglaa M. Kamal, Azza O. Elsheikh, Farida H. Omran, Ahmed Abdulsaboor, Maha M.H. Sakr, Jaber Alfaifi, Reem Turkistani, Futun Aljuaid, Mohammed A.M. Oshi.
